# Process mining framework with time perspective for understanding acute care: a case study of AIS in hospitals

**DOI:** 10.1186/s12911-021-01725-1

**Published:** 2021-12-19

**Authors:** Jianfei Pang, Haifeng Xu, Jun Ren, Jun Yang, Mei Li, Dan Lu, Dongsheng Zhao

**Affiliations:** 1 Information Center, Academy of Military Medical Sciences, Beijing, China; 2 Medical Service Department, General Hospital of Xinjiang Military Command, Urumchi, China; 3 China Stroke Data Center, Beijing, China; 4 Jiangsu 707 Natural Pharmaceutical Co., Ltd., Zhenjiang, China

**Keywords:** Acute care, Process mining, Ischemic stroke, Quality improvement, Visual analysis

## Abstract

**Background:**

Acute care for critical illness requires very strict treatment timeliness. However, healthcare providers usually cannot accurately figure out the causes of low efficiency in acute care process due to the lack of effective tools. Besides, it is difficult to compare or conformance processes from different patient groups.

**Methods:**

To solve these problems, we proposed a novel process mining framework with time perspective, which integrates four steps: standard activity construction, data extraction and filtering, iterative model discovery, and performance analysis.

**Results:**

It can visualize the execution of actual clinical activities hierarchically, evaluate the timeliness and identify bottlenecks in the treatment process. We take the acute ischemic stroke as a case study, and retrospectively reviewed 420 patients’ data from a large hospital. Then we discovered process models with timelines, and identified the main reasons for in-hospital delay.

**Conclusions:**

Experiment results demonstrate that the framework proposed could be a new way of drawing insights about hospitals’ clinical process, to help clinical institutions increase work efficiency and improve medical service.

## Background

Acute care for critical illness (such as AIS) requires strict treatment timeliness. It can lead to a patient’s death or disability without timely intervention [1]. Therefore, medical managers and physicians should accurately figure out the causes of low efficiency in acute care. However, critical illness treatment is a multi-disciplinary, collaborative and complex process, where multiple objectives need to be considered urgently and simultaneously [2]. These objectives, including the sequence of activities and minimizing waiting times, significantly impact medical quality and cost [3]. For example, clinical guidelines recommend that door-to-needle time (DNT) be within 60 min for intravenous thrombolysis therapy to improve outcomes effectively [4]. Due to the complexity of clinical business, few tools could compare the process differences between patient groups or medical institutions.

A series of key performance indicators (KPIs) related to acute care have been proposed [5]. These KPIs are useful to evaluate the medical quality, but they can only indicate service from some single point of view, lacking intuitive visualization of the whole process. For example, most medical institutions cannot determine whether the actual care process is consistent with their expectance. Besides, the calculation of KPIs often depends on the data collected manually, which increases the labor burden of medical staff.

At present, information systems such as Electronic Medical Record (EMR) have been widely used in hospitals and produce lots of log data. For example, many clinical activities including registration, consultation, exam, etc., will be performed during a patient’s admission. These log data reflect the actual situation of business participants, and could help gain a clear picture of the underlying clinical process. Process mining provides an opportunity to identify bottlenecks slowing down the efficiency in hospitals. By extracting knowledge from event logs, healthcare providers could discover, monitor and improve their business process [6, 7], such as analyzing performance in the emergency department [8], identifying bottlenecks and exploring solutions [9], obtaining the execution of alternative variants [10]. However, due to data heterogeneity in medical institutions, the process models discovered from event logs could be quite different, inhibiting the comparison consistently. Moreover, the control flow (i.e., the ordering of activities) can only express relative time relationships, lacking methods to express quantitative time dimensions directly.

Therefore, we introduce a process mining framework with time perspective for understanding acute care. Initially, we pay attention to the standardization for activities (events), which would help clinical staff observe and analyze the actual process generally. Based on event logs, the relevant KPIs can also be calculated automatically, improving the work efficiency of hospitals. To evaluate this framework, we analyze two groups of AIS patients from a large general hospital in China. We discover and visualize the common paths of treatment in process models with timelines, then recognize the process bottlenecks and explore some solutions. The remainder of this paper is organized as follows. Section II presents methods. The results of this case study are analyzed in Section III. Section IV discusses and Section V concludes the paper respectively.

## Methods

(All methods were carried out in accordance with relevant guidelines and regulations.)

**Fig. 1:**
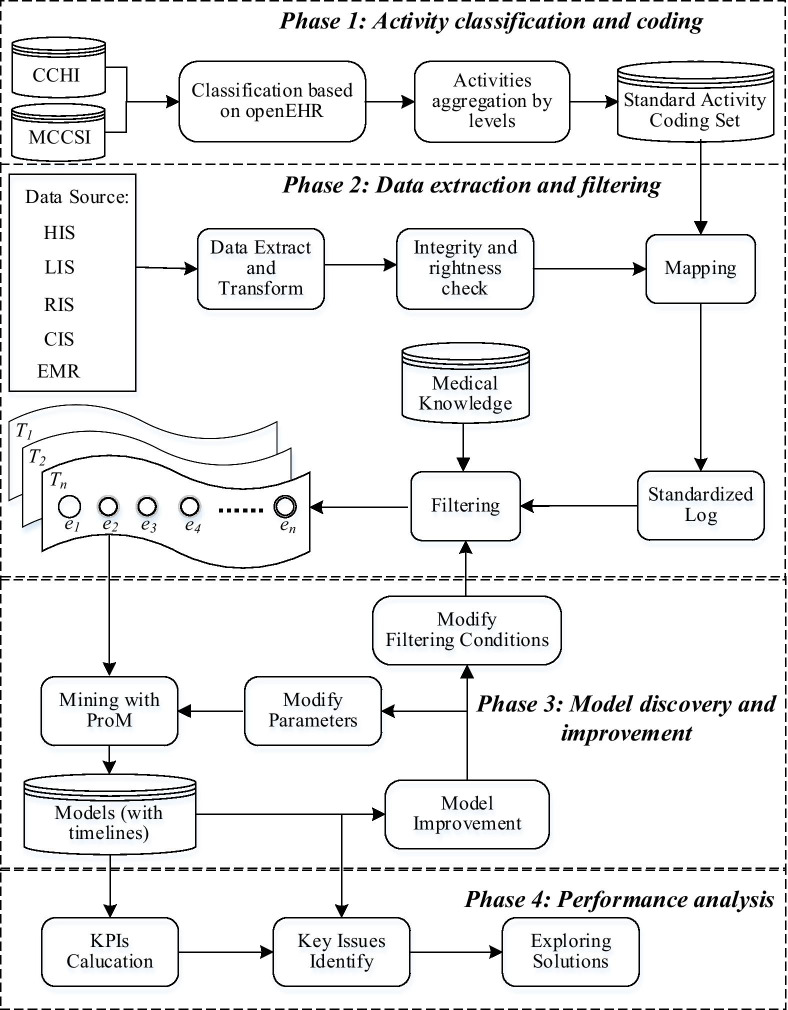
The process mining framework with time perspective

We propose a four-stage process mining framework with time perspective in Fig. 1. First, a standard activity set complied with openEHR is constructed. Second, patients’ data are extracted from multiple information systems and transformed into event logs, mapping with the standard activity set. After filtered by medical knowledge, patient traces in event log are import into ProM [11]. According to the clinical experts’ feedback, we modify parameters to generate and improve the process models with timelines iteratively. Finally, the KPIs are calculated, and possible key issues of in-hospital delay are identified in performance analysis.

### Activity classification and coding

**Fig. 2:**
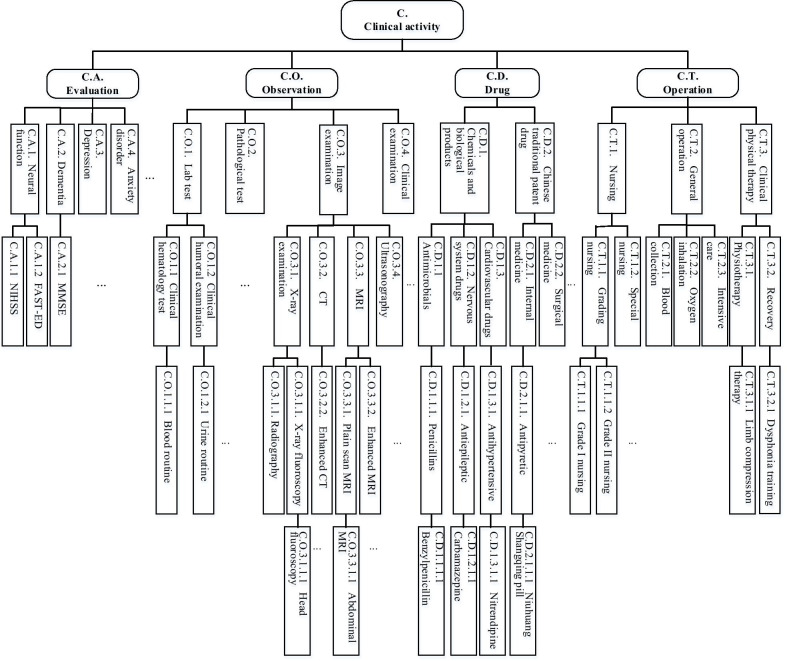
Classification and coding of clinical activities

In order to compare models consistently and abstract data at different levels, we construct a standard activity coding set. It can also solve data heterogeneity among different medical institutions to analyze their acute care process characteristics. First, we establish a hierarchical classification and coding system of clinical activities, shown in Fig. 2 (taken stroke as an example). To be understood and extended easily and to comply with international standards, we organize clinical activities by referring to the classification of openEHR archetypes [12]. The clinical events are divided into four categories: Evaluation, Observation, Drug and Operation. Since hospitals in China need to abide by national specifications, we subdivide each category by referring to the Chinese Classification of Health Interventions (CCHI) [13] and Medicine Classification and Codes for Social Insurance (MCCSI) [14]. CCHI could be applied to many business processes, such as medical record management, clinical research, and insurance payment. By now, CCHI contains 11392 medical service items, with an 8-digit code uniquely identifying each item. In this work, the subcategories of Evaluation, Observation and Operation are based on CCHI. For the drugs not covered by MCCSI, we classify them according to their pharmacological and functional indications. Therefore, the standard activity coding set is a multi-level classification system organized in a hierarchical tree structure, where the leaf nodes are the most specific. The closer to the root node, the more abstract it is.

Besides, we code the activities using a scalable form to ease management. For example, penicillin is coded as “C.D.1.1.1.1”, in which C represents clinical activity, D represents drug class, and “.” is used to distinguish different levels. When new nodes need to be added, the original classification architecture will not be affected in this coding system. For image examinations, the leaf node is a combination of examination type and a specific examination object. This is because the examination orders need to express the purpose explicitly. For example, “Head CT” is a combination of “CT examination” and “head.” Due to space limitation, only part of the clinical activities are shown in Fig. 2. For instance, “Penicillins” (C.D.1.1.1.) only show Benzylpenicillin, while amoxicillin and Piperacillin are not listed. The complete classification and coding of clinical activities for critical illness are shared in GitHub (https://github.com/asdwtwty/Classification-and-coding-of-clinical-activities).

### Data extraction and filtering

In this phase, the data for process mining is extracted from several information systems, including Hospital Information System (HIS), Clinical Information System (CIS), Radiology Information System (RIS), Laboratory Information System (LIS) and Electronic Medical Record (EMR). Structured Query Language (SQL) is used to capture the structured event data from database. Furthermore, we use Beautiful Soup [15], a Python library for natural language processing, to extract information from unstructured text. Besides, the integrity and correctness of the original event data are verified according to predefined rules. For example, if “onset time” and “arrival time” of one patient are extracted, then “onset time” should be earlier than “arrival time.” The complete rules in preprocessing stage to remove unreasonable patient data are shown in Table 1.

**Table 1:** The rules for preprocessing of patient data

No	Rules	Comments
1	The patient's onset time and arrival time cannot be missing	Exact minute
2	The patient's onset time is earlier than the arrival time	
3	The start time of orders is later than patient's arrival time	
4	Patient's arrival time is earlier than the time arrival of neurology department	Applicable to cooperative hospitals
5	If the patient has more than one thrombolytic drug order, we will choose the earliest order to determine the start time of thrombolytic therapy	Thrombolytic drugs include rt-PA and urokinase
6	The time of arrival of neurology department will be determined by the first order issued after admission	
7	Range of rt-PA dosage: 20 mg ≤ n ≤ 300 mg	
8	Range of door-to-needle time: 0 < n ≤ 360 min	
9	Range of time from onset to start of thrombolytic therapy: 0 < n ≤ 360 min	

Then event data are mapped to the standard activity set constructed in *Phase 1* to generate a standardized event log. Some related attributes will be appended in this mapping. For example, an event obtained from doctor's order “Head CT” represents the start event of “Head CT examination,” and the completion event is “the completion of Head CT examination.” Although each activity should contain the start and end events, it is difficult to obtain all the end events due to incomplete data records. In this study, we focus on the events (e.g., imaging exam, laboratory tests) that are important for thrombolytic therapy of stroke, defined in the clinical guidelines [16]. We extract the start and completion times of these activities from LIS and RIS. However, for less significant activities (such as nursing and physical examination) on thrombolytic therapy, since only the start time have been recorded in information systems, we assume that they started at the same time as they finished. Finally, the trace is obtained by arranging all events belonging to the same patient in ascending order. The standardized events data are stored in log files with eXtensible Event Stream (XES) [6] format.

If patient trace mapped by the atom activity set (leaf nodes in Fig. 2) are directly used for analysis and mining, the interpretability of process model may be poor because of too many types of events, that is, the “spaghetti effect” [17]. Therefore, we introduce a log filtering module before mining process models. The log filtering is based on existing medical knowledge for specific events or patients. The filtering conditions could be the time of event occurring or patients’ characteristics (demographic information, diagnosis, etc.). Events can also be abstracted based on the classification system shown in Fig. 2. The clustering operation aggregates the leaf nodes (activity) to their parent nodes, and users can customize the depth of aggregation. For example, the two drugs of “Nitrendipine” and “Nifedipine” could be aggregated into “antihypertensive drugs”, which can reduce the classes of events.

### Model discovery and improvement

Since the process models obtained may vary under different filtering conditions (e.g., discovery algorithms or hyperparameters), we use an iterative and incremental mining strategy to ensure the interpretability of models discovered. First, process models are discovered by iDHM [18], which is a plug-in of process mining tool ProM [11]. It can perform interactive process model discovery and exploration, integrating algorithms such as Alpha, Heuristics Miner and Fuzzy Miner. On the basis of Dependency graphs discovered, we add a time axis and rearrange all nodes in chronological order, which can display an intuitive graphic. In order to integrate and display multiple perspectives information, we use color, edge, and timeline to represent different dimensions. In other words, the five types of events are represented in boxes with different colors: Observation (green), Drug (red), Artificial events (black), Evaluation (orange), and Operation (blue). The edge between events represents the dependency relationship, and the value on it indicates the degree of dependence. For example, a dependency relation (*a*, *b*) means a causal dependency from activity *a* to activity *b*. Besides, a time axis is listed on the figure’s left side, illustrating the total duration of related events. Since the control flow in dependency graphs only reflects the qualitative relationship between clinical activities, we add extra temporal information for each event. The event occurrence time before arriving at hospital is negative, while it is positive for these events occurred after arriving at hospital. We compute a set of metrics such as the minimum, median, maximum time of events, and mark them after the event’s name. We also calculate the frequency ratio of events (event's frequency/number of patients in that group) to represent the process characteristic.

To overcome the misleading problem using Directly-Follows Graphs (DFG) in reflecting time perspective [19], we enhanced the traditional DFG models. The display time of each node (activity) is recalculated using our method. The time of “arrive at hospital” activity is taken as zero, and the median time from admission to each activity is used as the display time of that activity. The time between nodes can be seen directly from the time axis on the figure’s left side.

In this work, we established a team consist of three medical specialists, two informatics experts and one database engineer. After clinical experts evaluating the model’s interpretability, they work with the informatics experts to revise mining strategies (such as modified mining algorithms, log filtering conditions, etc.). Finally, the enhanced dependency graph models reflecting the actual clinical process are generated.

### Process analysis

After generating the process model, the KPIs (i.e., DNT) can be calculated objectively based on the log data. Hospital managers could compare these KPIs with the recommended values in clinical guidelines to determine key issues like in-hospital delay. Besides, they can compare the actual clinical pathways between hospitals, or different patient groups in the same institution. They can also analyze the causes of the problems combining with the process models, and figure out possible factors impacting treatment efficiency. For example, an analyst could find out the main clinical activities and orders in treatment of AIS, determining whether the actual clinical path is reasonable. Based on cause analysis for the issues concerned, hospital managers can put forward improvement suggestions or plans.

## Results

Stroke is the leading cause of death in China, which costs about 6 billion dollars each year according to the global burden of disease research [20]. In acute care stage, it requires the stroke unit (emergency physicians, neurologists, nurses, etc.) within a timely, high-quality treatment process to restore the supply of blood promptly [16, 21]. To verify our framework, the critical treatment process of AIS is chosen as a case study. We collected the EMR data of patients from multiple information systems of a large general hospital in China. All participants were diagnosed with AIS (ICD-10: I63, I64), and they did not receive endovascular therapy. For all the patients selected, the time from onset to consultation should be less than 6 h. The patients would be excluded if they were wake-up stroke or had received arterial thrombolysis or mechanical thrombectomy therapy. In this case study, a data set of 420 patients was selected, who had been admitted to the hospital from February 2013 to June 2019. In order to analyze the cause of DNT delay, these patients are divided into thrombolysis cohort (125) and non-thrombosis cohort (or conventional treatment cohort, 295) depending on whether they received intravenous thrombolysis therapy. Table 2 shows patients characteristic of the two cohorts. For example, the median onset time is 3.06 (±0.90) hours in the intravenous thrombolysis cohort, which is less than 4.3 (±1.4) hours in the non-thrombolysis cohort. More than 70% of patients are with hypertension, and about 30% have diabetes.

**Table 2:** Overview of thrombosis and non-thrombosis cohort

Characteristics	Intravenous thrombolysis (cohort = 125)	Non-thrombosis cohort (n = 295)
Mean (SD) age (years)	65.5 (13.1)	68.7 (13.3)
Men	78 (62.4%)	191 (64.7%)
Neurologic impairment*		
0	9 (7.2%)	50 (16.9%)
1	79 (63.2%)	164 (55.6%)
2	35 (28.0%)	79 (26.8%)
3	2 (1.6%)	2 (0.7%)
Hours (SD) from stroke onset	3.06 (0.90)	4.3 (1.4)
Medical history		
Stroke	4 (3.2%)	4 (1.4%)
Diabetes mellitus	39 (31.2%)	79 (26.8%)
Hypertension	95 (76%)	210 (71.2%)
Atrial fibrillation	19 (15.2%)	46 (15.6%)
Mean (SD) systolic blood Pressure (mm Hg)	145.1 (23.3)	143.1 (20.7)
Mean (SD) heart rate	76.9 (11.5)	78.9 (12.7)
Mean (SD) respiratory rate	16.8 (1.8)	17.1 (1.7)
Mean (SD) Temperature (℃)	36.5 (0.3)	36.4 (0.3)
Mean (SD) Glu (mmol/L)	8.2 (3.6)	7.9 (3.2)

*Neurologic impairment contains “Facial asymmetry”, “Arm and leg weakness”, “Slurring of speech”, adding one score for each positive

According to the activity classification system shown in Fig. 2, the data set collected involves 88 atomic activities. We distinguish in-hospital acute care into two stages: emergency and hospitalization (patients with AIS arrive at neurology department). Three artificial events (“onset,” “arrive at the hospital” and “arrive at the neurology department”) are added. In the standardized log, there are 124 event types. 121 of them are transformed from atomic activities, and 3 of them are artificial events. This study compares the treatment process between thrombosis and non-thrombosis cohort, to analyze whether there are in-hospital delay and their causes. In the thrombosis cohort, the last event of this process is “thrombolytic drug”, and the maximum DNT was 175 min (less than 3 h). However, in the non-thrombosis cohort, there is no obvious activity as the last event of this process. According to the median DNT time of thrombosis cohort (2 h) and intravenous infusion time of thrombolytic drug (1 h), we use their total time to estimate the process duration of non-thrombosis cohort. That is, the non-thrombosis cohort only includes those activities within 3 h after arriving at the hospital.

### Process model with timelines

**Fig. 3:**
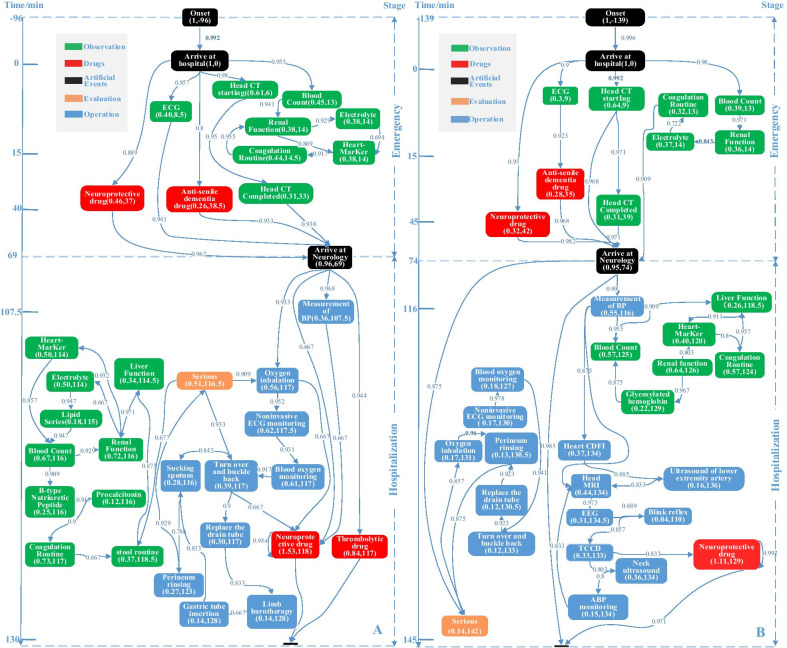
Process model discovered: Thrombolytic cohort (**A**), Non-thrombolytic cohort (**B**)

We apply the interactive process discovery strategy with medical experts, and modify the parameters (frequency, dependency, conditions, etc.) according to their opinions. After performed five iterations, Fig. 3 illustrates the process model (enhanced dependency graph) for in-hospital acute care of the two cohorts. The model discovered is consistent with clinical pathway of ischemic stroke issued by the National Health Commission of China. Moreover, the granularity of activities in our model is small, and each node contains activity name, frequency ratio and completion time. For example, the “ECG(0.40,8.5)”node in Fig. 3A indicates that it took 8.5 min from "Arrive at hospital" on average, and 40% of patients in the cohort performed this activity. We mark the median time of each event in the dependency graph model with timeliness. If a patient arrives at the hospital, the event occurrence time before arriving at hospital is negative, while the event occurrence time after arriving at hospital is positive.

In emergency stage, clinical activities and sequence of the two cohorts are basically the same. The main observation activities are ECG, CT and lab tests. The medicine mainly used are neuroprotective drugs (“Edaravone”, “Chinese traditional patent medicine”, etc.) and anti-dementia drugs (“Olacetam”). However, in hospitalization stage, the thrombolytic cohort carried out further lab tests, such as “Detection of myocardial ischemia markers”, “B-type natriuretic peptide”, etc. In this stage, the operation activities include all kinds of nursing activities, evaluation of patients' state, and some special treatment. The medicine includes “Neuroprotectivedrugs” and “Thrombolytic drugs.” Compared with the thrombolytic cohort, the non-thrombolytic cohort had more neurological and vascular examinations (e.g., “EEG”, “TCCD”, “MRI”).

For the intravenous thrombolysis cohort, the time interval between thrombolytic events and some other events is quite small, namely these events occur almost simultaneously. It is because neurologists issue relevant orders in batch on computers after necessary examinations were finished. Similar phenomena can be observed in the non-thrombolysis cohort. Perhaps, there is a need to issue bedside orders in time with the help of Personal Digital Assistant (PDA).

### Performance analysis

We can see that some lab tests in emergency and hospitalization stage are repeated (within 3 h of arriving at hospital). Confirmed by clinical staff, these repeated tests are mainly caused by the incompatibility of LIS between the emergency and hospitalization. In the hospital, neurologists cannot reprint the lab reports applied in emergency stage, while these reports are required according to treatment regulations. Doctors have to reapply some examination items after patients are admitted to the neurology department. The incompatibility of the information systems also increases patients’ economic burden. Therefore, it is suggested that the hospital upgrade information systems so that neurologists can easily review and print the result of tests applied in emergency stage. In Fig. 3, there is no event between arriving at neurology department and the next recorded operation (“blood pressure measurement”). In this phase, physician carries out a physical examination, asking medical history, talking with patient’s family members, and so on, while these activities’ time information is not recorded in the EMR system. The main KPIs used in care of AIS are shown in Table 3. The Imaging-to-Needle Time (INT) in this hospital is 33 min, close to the RCSN study in Canada [22]. However, compared with the SITS-MOST study [23], the DNT of this hospital is much longer (117 min vs. 68 min). Only 6.4% of patients received thrombolysis within 60 min, which is far behind the target value (75%) [24]. It indicates there is an in-hospital delay, which may occur between the imaging examination and thrombolysis therapy. There is also a large gap (37.1% vs. 71.6%) in the proportion of thrombolytic patients within 2 h. The patients’ median time from onset in thrombolytic cohort is significantly shorter than that in non-thrombolytic cohort (*P *< 0.05). On the other hand, there is no significant difference in the median time of completing head CT and arriving at neurology department (*P* > 0.05). Therefore, the timeliness between two cohorts in emergency stage is essentially the same.

**Table 3:** KPIs for AIS

KPIs	Case study result	Relevant researches result	Explanation
INT	33 min	31 min (RCSN in Canada)	Median
DNT	117 min	68 min (SITS-MOST)	Median
The proportion of patients with AIS received thrombolysis therapy within 60 min	6.4%	Target 75% (Report on Stroke Prevention and Treatment in China 2018)	Patients received intravenous thrombolysis therapy within 60 min after arrival hospital
Thrombolysis rate of patients arrive at hospital within 2 h	37.1%	71.6% (GWTG 2009 in the United States)	Patients who arrived within 2 h after initial symptom onset and were treated within 3 h

In Fig. 3A, although the INT is very short, the time before patient arrival at neurology department is over 60 min. The transit time (between “completion of Head CT examination” and “arriving at the neurology department”) is about 30 min. However, among the eight patients whose DNT is less than 60 min, seven of them stay less than 10 min in emergency department, suggesting that the early intervention of neurologists would shorten DNT. Therefore, the main reason for longer DNT in this hospital is that patients need to be transported to neurology department for thrombolytic therapy. In addition, patients with thrombolysis need to go through hospitalization procedures, which would also increase DNT. It is recommended that the hospital change thrombolytic site to emergency room to reduce transit time. At present, neurologists usually contact AIS patients after CT or other imaging examinations are finished, which would increase the DNT to a certain extent. Accordingly, it could be better if neurologists contact stroke patients earlier (e.g., when patients are suspected of stroke), and prepare the thrombolytic therapy procedures in advance.

## Discussion

The framework proposed in this study verifies that process mining can character clinical activities of acute care from time perspective. The medical staff who participated in this study affirmed the framework, especially for it intuitively characterize the time-critical medical procedures. It can automatically calculate the relevant KPIs, which is more efficient than the traditional time analysis methods. By comparing the process models of thrombolytic and non-thrombolytic cohorts, we discovered the cooperative hospital’s problems: unreasonable setting of thrombolysis sites and late participation of neurologists. We have reported the analysis results to the quality control department of cooperative hospital. After approval by the hospital managers, they decided to make an improvement plan based on the report. Compared with KPIs, the process models could objectively show the actual treatment path and provide more detailed information for analyzing acute care efficiency.

We also generated Petri Nets using ProM's Inductive Miner or Heuristic Miner. However, after discussion with medical experts, it is considered that the models are not easy to analyze the first aid process of thrombolysis, lacking intuitive display time and frequency dependence information between events. Perhaps there are few structured processes or too many noises in our event log, which is not suitable for Petri Nets. Therefore, the enhanced DFG model is used in practical application.

Process mining has been applied in healthcare, such as discovery of clinical process models [25], assessment of clinical process changes [26], finding role interactions [27] and answering posed questions [28]. The authors in [29] and [30] visualized the pre-hospitalization pathways and identified bottlenecks by dependency graph model, but the intravenous thrombolysis process was not involved. However, different from these studies, this paper expresses the time information clearly and intuitively, with a standardized data coding set to classify medical events. Cho et al. [31] used an approach named process improvement (PI) to reduce the in-hospital delay by monitoring a key indicator (the time interval from emergency department arrival to intravenous thrombolysis). However, getting KPIs to evaluate stroke emergency efficiency usually requires manual collection of relevant information, and their evaluation ability often depends on experts’ experience.

In China, many hospitals have established first-aid centers that integrate medical resources to achieve efficient and standardized critical illness treatment. Some hospitals also have opened the “green channel” in emergency service for stroke patients, a multi-disciplinary integrated treatment pattern, to further shorten the time of clinical processes [22]. To build the emergency center or “green channel”, medical institutions should optimize the whole process of treatment. A data-based tool that comprehensively and objectively reflects the actual treatment process is needed, to support medical quality’s continuous improvement.

The contribution of this study as follows. Firstly, based on openEHR, CCHI and MCCSI, we construct a common data classification and coding set for clinical activities, supporting multi-level mapping and processes comparison between institutions. Secondly, it provides a method to enhance process mining with expression from the qualitative and quantitative time perspective.

Although this study takes the acute care of ischemic stroke as an example, the presented methodology could be extended to other time-critical diseases like ST-segment elevation myocardial infarction (STEMI) [32]. It illustrates a practical approach for drawing insights into the clinical process. The KPIs and issues identified (e.g., in-hospital delay) can be visualized in a fine-grained and standardized way. Furthermore, based on the process model generated by information technology staff, medical experts can analyze and propose improvement suggestions. This kind of cooperation can also be promoted in other data-driven projects.

## Conclusions

This study proposed a novel process mining framework with a common data set to analyze acute care from time perspective. We find that process mining can intuitively characterize the time-critical medical procedures, helping to analyze the causes of delay and evaluate the existing treatment process. This data-based method can also help medical institutions to compare and optimize the acute care procedure to achieve “timeliness, accuracy and efficiency” targets. There are also limitations in this study: the results are based on the data from only one hospital, and some medical activities are not recorded completely. In the future, more clinical data in the acute care process (especially from multiple medical institutions) would be collected to verify the effectiveness of this method.

## Data Availability

The datasets used and/or analyzed during the current study are available from the corresponding author on reasonable request.

## Abbreviations

AIS: Acute ischemic stroke
KPI: Key performance indicator
DNT: Door-to-needle time
EMR: Electronic medical record
CCHI: Chinese classification of health interventions
MCCSI: Medicine classification and codes for social insurance
HIS: Hospital information system
CIS: Clinical information system
RIS: Radiology information system
LIS: Laboratory information system
SQL: Structured query language
ICD: International classification of diseases
ECG: Electrocardiogram
EEG: Electroencephalogram
TCCD: Transcranial color-coded duplex sonography
CT: Computed tomography
MRI: Magnetic resonance imaging
PDA: Personal digital assistant
INT: Imaging-to-needle time
iDHM: Interactive data-aware heuristic miner
